# Organochlorine Pesticides and Male Genital Anomalies in the Child Health and Development Studies

**DOI:** 10.1289/ehp.7382

**Published:** 2004-11-04

**Authors:** Rajiv Bhatia, Rita Shiau, Myrto Petreas, June M. Weintraub, Lili Farhang, Brenda Eskenazi

**Affiliations:** ^1^San Francisco Department of Public Health, San Francisco, California, USA; ^2^Hazardous Materials Laboratory, Department of Toxic Substances Control, California Environmental Protection Agency, Berkeley, California, USA; ^3^Public Health Institute, Berkeley, California, USA; ^4^Center for Children’s Environmental Health Research, School of Public Health, University of California at Berkeley, Berkeley, California, USA

**Keywords:** cryptorchidism, DDE, DDT, hypospadias, insecticides, male genital anomalies, organochlorine, pregnancy

## Abstract

Increasing rates of cryptorchidism and hypospadias in human populations may be caused by exogenous environmental agents. We conducted a case–control study of serum levels of *p*,*p*′-dichlorodiphenyltrichloroethane (DDT) and its major metabolite, *p*,*p*′-dichlorodiphenyldichloroethylene (DDE), and cryptorchidism and hypospadias in the Child Health and Development Study, a longitudinal cohort of pregnancies that occurred between 1959 and 1967, a period when DDT was produced and used in the United States. Serum was available from the mothers of 75 male children born with cryptorchidism, 66 with hypospadias, and 4 with both conditions. We randomly selected 283 controls from the cohort of women whose male babies were born without either of these conditions. Overall, we observed no statistically significant relationships or trends between outcomes and serum measures. After adjusting for maternal race, triglyceride level, and cholesterol level, compared with boys whose mothers had serum DDE levels < 27.0 ng/mL, boys whose mothers had serum DDE levels ≥61.0 ng/mL had odds ratios of 1.34 [95% confidence interval (CI), 0.51–3.48] for cryptorchidism and 1.18 (95% CI, 0.46–3.02) for hypospadias. For DDT, compared with boys whose mothers had serum DDT levels < 10.0 ng/mL, boys whose mothers had serum DDT levels ≥20.0 ng/mL had adjusted odds ratios of 1.01 (95% CI, 0.44–2.28) for cryptorchidism and 0.79 (95% CI, 0.33–1.89) for hypospadias. This study does not support an association of DDT or DDE and hypospadias or cryptorchidism.

Hypospadias (an abnormal opening of the urethra) and cryptorchidism (a failure of one or both testicles to descend) are two relatively common male genital congenital anomalies, occurring in 35 of 10,000 and 40 of 10,000 births, respectively (Paulozzi 1999). Recent evidence has suggested that hypospadias and cryptorchidism have increased in frequency in some populations (Paulozzi 1999). This is of public health significance, particularly because of the well-known association between cryptorchidism and testicular cancer (Wilson and Foster 1985; Toppari et al. 1996), which also appears to be on the increase (Toppari et al. 1996).

In mammals, cryptorchidism depends on mullerian-inhibiting hormone, androgens, and intra-abdominal pressure (Wilson and Foster 1985). The formation of the male external genitalia *in utero* also occurs under the influence of androgens (Baskin et al. 2001). There is suggestion that increasing rates of hypospadias and cryptorchidism may be caused partly by early exposure to endocrine-disrupting chemicals in the environment (Beard et al. 1984; Berkowitz et al. 1995; Depue 1984; Hjertkvist et al. 1989; Jackson 1988; Landrigan et al. 2003; McBride et al. 1991; Sharpe and Skakkebaek 1993; Sweet et al. 1974; Swerdlow et al. 1983; Toppari et al. 1996). This hypothesis is supported by the higher rates of urogenital anomalies, including cryptorchidism, in studies of sons exposed *in utero* to diethylstilbestrol, a potent estrogen (Cosgrove et al. 1977; Gill et al. 1979).

Although the use of one potent environmental endocrine disruptor, dichlorodiphenyl-trichloroethane (DDT), has been banned in the United States since 1972, concerns about its potential health effects continue because of its worldwide use in the eradication of malaria, its accumulation in the human food chain, and its long half-life in human tissues due to its lipophilicity (Clarkson 1995). DDT functions as an exogenous estrogen (Bulger and Kupfer 1983) and, in animal studies, has been shown to suppress Leydig cell development, alter secretion of mullerian-inhibiting substance by the Sertoli cells, and cause negative feedback inhibition at the fetal pituitary gland (Sharpe and Skakkebaek 1993). *p*,*p*′-Dichlorodiphenyl-dichloroethylene (DDE), the persistent metabolite of *p*,*p*′-dichlorodiphenyltrichloroethane (DDT), acts as an androgen receptor antagonist at target tissues and has been shown to inhibit the action of testosterone (Danzo 1997; Kelce et al. 1995). DDT or DDE may alter sex hormone metabolism, reducing available testosterone to tissues (Guillette et al. 1995). Adverse reproductive system effects associated with *in utero* DDT or DDE exposure in male animals include abnormal development of ovarian tissue (Fry and Toone 1981), reduced penis size (Guillette and Guillette 1996), reduced testosterone levels (Subramanian et al. 1987), reduced male rat anogenital distance (Gray et al. 2001), hypospadias (Gray et al. 2001), cryptorchidism (Facemire et al. 1995; Gray et al. 2001), impaired reproductive capacity (Bowerman et al. 1995), low sperm density (Facemire et al. 1995), and abnormal sperm (Facemire et al. 1995).

Few epidemiologic studies provide information on the relationship between male reproductive disorders and organochlorine insecticides in humans. García-Rodríguez et al. (1996) found higher rates of orchidopexy (a surgical procedure to correct cryptorchidism) in districts in Spain with more intensive farming. However, Longnecker et al. (2002) found no clear evidence of an effect of DDE on hypospadias or cryptorchidism among subjects in the Collaborative Perinatal Project. We report on an analysis of serum levels of DDT and DDE in a nested case–control study of cryptorchidism and hypospadias in a longitudinal cohort of pregnancies that occurred between 1959 and 1967, a period when DDT was produced and used in the United States but before the beginning of the rise in prevalence of hypospadias in the United States (Baskin et al. 2001).

## Materials and Methods

The Child Health and Development Studies (CHDS) is a longitudinal cohort study of 20,754 pregnancies among women enrolled in the San Francisco Bay Area Kaiser Foundation Health Plan between 1959 and 1967 (van den Berg et al. 1988). Nearly 92% of all eligible pregnancies invited to participate were included in the cohort. Subscribers to this prepaid health plan represented an economically and ethnically diverse urban population.

Pregnant women were interviewed shortly after recruitment and later during their pregnancies. Maternal and pediatric medical records were abstracted throughout the follow-up period. Abstracted records contained information from every child visit until at least 5 years of age, including physician’s diagnosis and treatment, test results, and anthropometric measures (Christianson et al. 1981). Children averaged at least three visits per year from birth until 5 years of age. The study’s rate of attrition was extremely low; the CHDS observed 89.4% of live-born children until 5 years of age.

The ascertainment of congenital anomalies occurred through routine medical procedures and practices and appropriate specialized tests and referrals. The CHDS itself did not implement any additional routine or special tests to ascertain congenital anomalies. At least two physicians and one biostatistician reviewed abstracted information to ensure uniform recording. Congenital anomalies were classified based on the *International Classification of Diseases, 7th Revision* [World Health Organization (WHO) 1957]. An anomaly was coded as definite only if a physician considered the diagnosis certain or if confirmed by surgery or laboratory tests. Abstracted data were subsequently computer coded (Christianson et al. 1981).

Blood samples were obtained from the pregnant women at the time of enrollment, during each subsequent trimester, and immediately after delivery. At least one sample was obtained for 89% of pregnancies. Serum samples were subsequently divided into a package of four 2-mL vials and stored at −20°C at the National Institutes of Health (Bethesda, MD).

For this nested case–control study, we used a subset of males who were followed by the CHDS for at least 2 years and for whom computer records showed that at least one serum sample had been collected and stored. We restricted the subset to this minimum length of follow-up because cryptorchidism was coded by CHDS as an anomaly only if it persisted until 2 years of age.

Among the 9,345 males followed until 2 years of age, 101 had cryptorchidism, 73 had hypospadias, and 6 had both. We were able to obtain serum from the mothers of 75 subjects with cryptorchidism, 66 subjects with hypospadias, and 4 subjects with both conditions. We randomly selected 283 controls from the remaining male singleton births who were followed to 2 years of age, did not have hypospadias or cryptorchidism, and had one recorded serum sample. There were no matching criteria for controls.

### Laboratory assays.

If available, the serum conservators provided the last pregnancy serum of the mother (*n* = 86); otherwise, samples from the postpartum period were provided (*n* = 334). Given the long half-life of DDT and DDE and the high correlation among DDE levels measured at different times during gestation (Longnecker et al. 1999), these serum samples should accurately reflect body burdens over the entire pregnancy. National Cancer Institute staff in Frederick, Maryland, retrieved the requested archived serum, placed a 1.5-mL aliquot of the sample into a separate vial, assigned a study identification number, and shipped the samples overnight on dry ice to the Hazardous Materials Laboratory of the State of California in Berkeley, California, where they were stored at below −20°C until laboratory analysis. Standard quality assurance procedures included rigorous calibration procedures, traceability of all standards, and internal review and audit. Method (reagent) blanks and laboratory controls were performed on either 10% of the samples or at least one with every batch of samples, whichever was greater. Internal standard recovery was performed on every chemical group on every sample. The laboratory staff were blind to the identity of the samples and to case/control status.

Laboratory personnel performed analyses in batches of 12 samples. Each batch consisted of nine study subject samples, one method blank, one laboratory control (fortified bovine serum), and a standard reference material [SRM 1589a, a human serum from the National Institute of Standards and Technology (Gaithersburg, MD)]. Batches included a consistent proportion of cases and controls. Unbeknown to laboratory staff, some batches included samples of pooled CHDS serum in place of a study subject sample to assess performance and to also facilitate future interlaboratory standardization.

Analytical methods are described in detail elsewhere (Petreas et al. 2003). Briefly, serum was thawed, and 1 mL was pipetted into a 15-mL test tube. Internal standards [polychlorinated biphenyl (PCB) congeners 14, 65, and 166 and tetrachloromethyl xylene (TCMX; AccuStandard, Inc., New Haven, CT)] were added before denaturing the proteins with 1 mL of acetic acid (Fisher Scientific, Pittsburgh, PA). Solvents employed were nanograde isooctane (Mallinckrodt, Paris, KY), trace environmental analysis grade hexane (99.9%), methanol (99.9%), dichloromethane (99.9%), pesticide residue grade acetone (99.9%), and toluene (99.9%) (Burdick & Jackson, Muskegon, MI). The analytes in the serum were then extracted with hexane:dichloromethane (90:10, vol:vol), and the extract was passed through a glass column filled with Florisil. The analytes were eluted with hexane followed by hexane: dichloromethane (1:1, vol:vol). The eluates were combined and concentrated, and recovery standards (pentachloronitrobenzene, PCB-30, PCB-204, and PCB-209) were added. We used six-level calibration curves with concentrations encompassing expected ranges for each analyte. Analysis was performed by gas chromatography/electron capture detection (Hewlett Packard 6890; Agilent Technologies, Palo Alto, CA) equipped with 60-m DB-XLB (Agilent Technologies) and Rtx-5ms capillary gas chromatography columns (Resick Corporation, Bellefonte, PA). Total lipids were calculated from total cholesterol and triglycerides (Phillips et al. 1989). We determined total cholesterol and triglycerides enzymatically in a small aliquot of serum at the Clinical and Epidemiological Research Laboratory, Boston Children’s Hospital (Boston, MA), and results were reported both as nanograms per milliliter of serum and as nanograms per gram lipid.

We used recoveries of internal standards (PCB-14, PCB-65, PCB-166 and TCMX) to gauge overall data quality for all analytes across all serum batches. Recoveries were between 81 and 99%, and no corrections were made to the measurements. Control charts on the performance of the laboratory controls (reagent blanks, fortified bovine serum, and SRM 1589a) were maintained for all analytes across all batches to ensure that results were within quality control (QC) criteria. Of the samples analyzed, 420 were from participants and 20 were blind laboratory controls interspersed among the actual samples serving as external QC controls. The identity of the 20 external QC controls was revealed only at the end of the analyses, and results were used to assess precision among batches. Based on these external QC samples, within-batch precision [expressed as the intrabatch coefficient of variation (CV%)] was 2.71% for DDT and 3.01% for DDE. The interbatch CV% was 9.97% for DDT and 9.11% for DDE.

### Statistical methods.

Preliminary analyses involved univariate examination of serum measures and covariates of interest using summary statistics. Among the available CHDS information, we selected variables known from the literature to be related to exposures or outcomes as potential covariates: maternal age, prepregnancy body mass index (BMI), parity, maternal ethnicity, maternal place of birth, maternal occupation before pregnancy, birth weight, gestational age, date of blood draw, and season of birth. We plotted the cumulative distribution as well as density and quintile plots. We performed bivariate analysis of covariates and case/control status using logistic regression. We performed bivariate analysis between covariates and the distribution of serum measures using linear regression for continuous variables and analysis of variance for categorical variables. Because 25% of all observations were missing either height or prepregnancy weight, we imputed the prepregnancy BMI for those women who were missing only prepregnancy weight by calculating median weight gained during pregnancy for women in each pregnancy BMI category and applied this weight change to the women whose weight was measured at the same point during their pregnancy.

We examined the relationship between exposure and case status for both cryptorchidism and hypospadias using logistic regression. For models that included serum measures as continuous variables, we used log-transformed values. For categorical analysis, we identified quartile cut points based on the distribution of each measure among the whole study sample. All regression analyses included cholesterol and triglycerides (milligrams per deciliter serum) as separate continuous variables.

We included all covariates in a backward stepwise elimination model (exclusion criteria *p* < 0.20) to evaluate the influence of these potential confounders on the effect of exposures to DDT or DDE on cryptorchidism and hypospadias. Separate models were evaluated for cryptorchidism and for hypospadias; models for all cases combined were also examined. Finally, we assessed the effect of the ratio of DDE to DDT on the outcomes as a way to evaluate the effect of recentness of exposure to DDT. All analyses were performed using the open source statistical program RGui, version 1.3.1 (R Foundation for Statistical Computing, Vienna, Austria).

## Results

Most maternal, child, and delivery characteristics examined were similar among cases and controls (Table 1); a higher proportion of hypospadias cases were born to white mothers, and mothers with offspring with cryptorchidism had higher prepregnancy BMI than did control mothers. Mean DDE and DDT levels, whether expressed as a serum concentration (nanograms per milliliter serum) or lipid adjusted (micrograms per gram lipid), did not differ among cryptorchidism cases, hypospadias cases, and controls (Table 2).

## Discussion

Our analysis did not find a statistically significant adverse association between maternal serum measures of DDT or DDE and cryptorchidism or hypospadias among pregnancies enrolled in the CHDS in California in the 1960s, when levels of exposure were considerably higher than they are today. The results of our study are consistent with those reported on hypospadias and cryptorchidism and DDE in archived maternal serum from the Collaborative Perinatal Project, a study conducted concurrently with the CHDS. The Collaborative Perinatal Project found that after adjusting for maternal race, triglyceride level, and cholesterol level, compared with boys whose mothers had serum DDE levels in the lowest quintile (< 21.4 ng/mL), boys whose mothers had serum DDE levels in the highest quintile (≥85.6 ng/mL) had ORs of 1.3 (95% CI, 0.7–2.4) for cryptorchidism and 1.2 (95% CI, 0.6–2.4) for hypospadias (Longnecker et al. 2002). Although our cut points differed from those in the Collaborative Perinatal Project, our results were comparable, with adjusted OR comparing boys whose mothers had serum DDE levels in the highest quartile (≥61.0 ng/mL) to boys whose mothers had serum DDE levels in the lowest quartile (< 27.0 ng/mL) at 1.34 (95% CI, 0.51–3.48) for cryptorchidism and 1.18 (95% CI, 0.46–3.02) for hypospadias. The similarity in the results between these two studies was found despite differences in the geographic location of the subjects (northern California vs. 12 centers across the United States), participants’ health care services (prepaid health plan vs. university-based practice), and case inclusion criteria (cryptorchidism present after 2 years of age vs. cryptorchidism diagnosed in first year of life).

Our study had several strengths. It sampled a population from a large prospective cohort study undertaken at a single site with excellent subject retention and reliable information on a number of relevant covariates. Only two subjects in our sample had used hormones in the interval from 6 months before the last menstrual period up to the pregnancy, so the sample was not subject to bias because of the effects of hormone use.

The study had consistent procedures for identifying and confirming congenital anomalies. Testicular descent is a dynamic process in that the prevalence of undescended testes may decrease with age (John Radcliffe Hospital Cryptorchidism Study Group 1992). Unlike the Collaborative Perinatal Project (Longnecker et al. 2002), the CHDS coded cases of cryptorchidism only if observed for 2 years, which allowed for spontaneous testicular descent and decreased the possibility of case misclassification. However, we could not exclude cases of cryptorchidism first observed after 1 year of age, which may have lead to a misdiagnosis of “retractile testes.” Nevertheless, the prevalence of cryptorchidism in the CHDS and Collaborative Perinatal Project studies was identical (1.08%), although the prevalence of hypospadias in the Collaborative Perinatal Project was about 25% higher (0.96 vs. 0.78%). Any misclassification of cases would be nondifferential with respect to exposure and would therefore attenuate any findings of an association.

The CHDS enrolled subjects at a time of high U.S. use of organochlorine insecticides (Kutz et al. 1991). Serum measures of DDE found in this study (43 ng/mL or 5.2 μg/g lipid) are comparable with those estimated from samples of body fat during the late 1960s and are among the highest recorded in U.S. populations (Kutz et al. 1991). Our results are comparable with measures of DDE (54 ng/mL or 6.9 μg/g lipid) from another analysis of CHDS archived maternal serum (James et al. 2002) as well as with those (43 ng/mL) found in an analysis of archived serum collected between 1964 and 1971 in northern California (Krieger et al. 1994). Notably, the CHDS mothers had DDE levels that were higher than the recovery-adjusted DDE levels in the Collaborative Perinatal Project mothers (34.3 ng/mL and 4.24 μg/g lipid) (Longnecker et al. 2002).

Serum measures in this population for DDE are also comparable with those associated with reproductive effects seen in eagles in their natural environments. In a study of nestling eagles, Bowerman et al. (1995) showed that productivity, measured as the number of young eagles observed in occupied nests, varied inversely with DDE levels measured in plasma of the same populations of eagles, which ranged from 5 to 40 ng/g. The serum DDE levels in our study are sufficient to inhibit androgen activity, based on *in vitro* effects. Kelce et al. (1995) found that 63.6 ng/mL DDE was sufficient to inhibit androgen receptor transcriptional activity *in vitro.*

Our study was limited in that serum was not available for all cases in the cohort, and this could have resulted in a bias. If the missing cases all had high DDT or DDE levels, then selection bias could have caused us to miss a positive association. An unmeasured confounder could also explain these results if levels of DDT or DDE varied with a protective factor for hypospadias or cryptorchidism. For example, fish consumption may promote fetal growth, a possible protective factor against the disorders studied, and may be associated with higher human cumulative exposure to organochlorines (Olsen and Secher 2002; Olsen et al. 1990, 1993). Notably, one U.S. study found no relationship between dietary consumption of fish and serum DDE levels (Laden et al. 1999).

Few models exist to adequately examine the combined effects of multiple chemicals with potential endocrine activity. Competing effects due to environmental agents with similar exposure pathways may obscure relationships between cause and effect in epidemiologic studies. For example, an androgen antagonist may oppose the effects of an estrogen agonist on the human pituitary because both androgens and estrogens inhibit luteinizing hormone secretion. Similarly, if DDE and DDT have differing effects, they could oppose each other and obscure any direct associations with adverse outcomes.

In summary, our study does not provide epidemiologic support for a causal adverse relationship between DDT or DDE and cryptorchidism or hypospadias. Our sample size was adequate to identify an approximate doubling of risk for the outcomes under study relative to the range of serum measures; however, the study may lack sufficient power to find a more modest effect with the observed exposure levels. Overall, DDT and DDE remain appropriate candidates for the study of environmental endocrine effects, especially given their wide use, their environmental persistence, their documented adverse reproductive effects in animals, and effects on other reproductive outcomes. Although this study does not support an association of DDT or DDE and hypospadias and cryptorchidism, the continued use of DDT in vector control in developing countries and its global distribution warrant further inquiry.

## Figures and Tables

**Table 1:** Characteristics of mothers and male offspring in a nested case–control study of U.S. participants in the CHDS, 1959–1967.

Characteristics	Cryptorchidism cases (*n* = 75)	Hypospadias cases (*n* = 66)	Controls (*n* = 283)
Maternal characteristics			
Mean ± SD [age (years)]	27.9 ± 6.5	26.4 ± 6.0	26.6 ± 6.2
Race [no. (%)]			
White	46 (61.3)	49 (74.2)	173 (61.1)
Latino	1 (1.3)	1 (1.5)	9 (3.2)
Black	21 (28.0)	9 (13.6)	82 (29.0)
Asian	5 (6.7)	4 (6.1)	10 (3.5)
Other	1 (1.3)	1 (1.5)	7 (2.5)
Unknown	1 (1.3)	2 (3.0)	2 (0.7)
Highest level of education completed [no. (%)]			
< 12th grade	14 (18.7)	9 (13.6)	50 (17.7)
High school graduate/trade school	44 (58.7)	37 (56.0)	160 (56.5)
College graduate	13 (17.3)	10 (15.2)	38 (13.4)
Unknown	4 (5.3)	10 (15.2)	35 (12.4)
Household income [no. (%)]			
< $5,000	15 (20.0)	10 (15.2)	45 (15.9)
$5,000–9,999	32 (42.7)	31 (47.0)	119 (42.0)
$10,000–14,999	10 (13.3)	9 (13.6)	38 (13.4)
≥$15,000	2 (2.7)	1 (1.5)	3 (1.1)
Unknown	16 (21.4)	15 (22.8)	78 (27.5)
Ever smoked [no. (%)]			
Yes	31 (41.3)	28 (42.4)	117 (41.3)
No	36 (48.0)	27 (40.9)	111 (39.2)
Unknown	8 (10.7)	11 (16.7)	55 (19.4)
Years lived on a farm before age 15 [no. (%)]			
None	37 (49.3)	39 (59.1)	142 (50.2)
1–4	5 (6.7)	6 (9.1)	19 (6.7)
≥5	14 (18.7)	4 (6.1)	34 (12.0)
Unknown	19 (25.3)	17 (25.8)	88 (31.1)
Maternal place of birth [no. (%)]			
California	30 (40.0)	24 (36.4)	85 (30.0)
Southeastern United States	15 (20.0)	7 (10.6)	66 (23.3)
Other U.S. states	20 (26.7)	18 (27.3)	73 (25.8)
Non-United States	6 (8.0)	7 (10.6)	24 (8.5)
Unknown	4 (5.3)	10 (15.2)	35 (12.4)
Parity [no. (%)]			
0	24 (32.0)	22 (33.3)	84 (29.7)
1–2	29 (38.7)	30 (45.5)	113 (39.9)
≥3	21 (28.0)	12 (18.2)	78 (27.6)
Unknown	1 (1.3)	2 (3.0)	8 (2.8)
Median [IQR] prepregnancy BMI (kg/m^2^)	22 [20–25]	21 [20–24]	21 [20–24]
Median [IQR] age at menarche (years)	13 [12–13]	12 [11–13]	12 [11–13]
Child’s characteristics			
Season of birth [no. (%)]			
January–March	17 (22.7)	23 (34.8)	65 (23.0)
April–June	20 (26.7)	12 (18.2)	68 (24.0)
July–September	22 (29.3)	18 (27.3)	70 (24.7)
October–December	16 (21.3)	13 (19.7)	80 (28.3)
Method of delivery [no. (%)]			
Vaginal	74 (98.7)	62 (93.9)	274 (96.8)
Cesarian	1 (1.3)	4 (6.1)	9 (3.2)
Small for gestational age [no. (%)]	6 (8.0)	9 (13.6)	36 (12.7)
Preterm birth [no. (%)]	2 (2.7)	3 (4.5)	21 (7.4)
Median [IQR] gestational age (weeks)	40 [39–41]	40 [38–41]	40 [29–41]
Median [IQR] birth weight (g)	3,374 [3,048–3,671]	3,260 [2,948–3,622]	3,345 [3,005–3,657]

IQR, interquartile range.

**Table 2:** Serum concentration distributions for organochlorine compounds in a nested case–control study of U.S. participants in the CHDS, 1959–1967.

Serum measures	Cryptorchidism cases [*n* = 75; median (IQR)]	Hypospadias cases [*n* = 66; median (IQR)]	Controls [*n* = 283; median (IQR)]
DDE serum concentration (ng/mL)	43.0 (32.0–60.0)	41.0 (30.2–57.8)	43.0 (32.0–56.5)
DDE, lipid adjusted (μg/g lipid)	5.3 (3.9–7.3)	4.6 (3.5–6.6)	5.2 (3.8–6.9)
DDT serum concentration (ng/mL)	12.1 (8.7–18.1)	9.5 (7.5–14.2)	11.1 (8.4–16.1)
DDT, lipid adjusted (μg/g lipid)	1.4 (1.0–2.0)	1.2 (0.9–1.6)	1.4 (1.0–1.9)
Serum cholesterol concentration (g/L)	2.5 (2.1–3.0)	2.6 (2.2–3.0)	2.5 (2.1–3.0)
Serum triglycerides concentration (g/L)	1.7 (1.4–2.4)	2.1 (1.5–2.6)	1.8 (1.4–2.3)
Total serum lipid concentration (g/L)	8.0 (6.7–9.5)	8.6 (7.3–9.9)	8.1 (7.0–9.5)

IQR, interquartile range.

**Table 3:** Adjusted ORs (95% CI) for birth defects among male offspring by DDE level in mother’s serum, CHDS, 1959–1967.

DDE (ng/mL serum)	No. of Cases	No. of Controls	Adjusted*^a^* OR (95% CI)	Adjusted*^b^* OR (95% CI)
All cases				
< 27.0	21	42	Reference	Reference
27.0–43.9	53	107	0.95 (0.50–1.77)	0.99 (0.52–1.89)
44.0–60.9	35	83	0.79 (0.41–1.55)	0.86 (0.43–1.70)
≥61.0	28	51	1.02 (0.50–2.09)	1.24 (0.58–2.63)
*p*-Value for trend			0.89	0.72
Cryptorchidism				
< 27.0	10	42	Reference	Reference
27.0–43.9	30	107	1.16 (0.52–2.60)	1.17 (0.51–2.66)
44.0–60.9	19	83	0.94 (0.40–2.24)	0.95 (0.39–2.30)
≥61.0	16	51	1.29 (0.52–3.22)	1.34 (0.51–3.48)
*p*-Value for trend			0.77	0.75
Hypospadias				
< 27.0	12	42	Reference	Reference
27.0–43.9	24	107	0.73 (0.33–1.62)	0.81 (0.36–1.84)
44.0–60.9	16	83	0.61 (0.26–1.43)	0.68 (0.28–1.64)
≥61.0	14	51	0.86 (0.35–2.10)	1.18 (0.46–3.02)
*p*-Value for trend			0.7	0.82

a Adjusted for cholesterol and triglyceride levels.

b Adjusted for cholesterol level, triglyceride level, and maternal race.

**Table 4:** Adjusted ORs (95% CI) for birth defects among male offspring by DDT level in mother’s serum, CHDS, 1959–1967.

DDE (ng/mL serum)	No. of Cases	No. of Controls	Adjusted*^a^* OR (95% CI)	Adjusted*^b^* OR (95% CI)
All cases				
< 10.0	65	117	Reference	Reference
10.0–14.9	29	87	0.59 (0.35–0.99)	0.63 (0.37–1.07)
15.0–19.9	24	37	1.13 (0.62–2.06)	1.25 (0.66–2.36)
≥20.0	19	42	0.77 (0.41–1.44)	0.89 (0.46–1.72)
*p*-Value for trend			0.62	0.98
Cryptorchidism				
< 10.0	32	117	Reference	Reference
10.0–14.9	12	87	0.50 (0.24–1.02)	0.49 (0.23–1.01)
15.0–19.9	20	37	1.95 (0.99–3.83)	2.04 (1.00–4.18)
≥20.0	11	42	0.95 (0.43–2.07)	1.01 (0.44–2.28)
*p*-Value for trend			0.42	0.38
Hypospadias				
< 10.0	34	117	Reference	Reference
10.0–14.9	18	87	0.70 (0.37–1.32)	0.81 (0.42–1.56)
15.0–19.9	5	37	0.45 (0.16–1.24)	0.45 (0.16–1.28)
≥20.0	9	42	0.66 (0.28–1.52)	0.79 (0.33–1.89)
*p*-Value for trend			0.15	0.30

a Adjusted for cholesterol and triglyceride levels.

b Adjusted for cholesterol level, triglyceride level, and maternal race.
